# Research progress of natural products targeting tumor-associated macrophages in antitumor immunity: A review

**DOI:** 10.1097/MD.0000000000040576

**Published:** 2024-11-15

**Authors:** Wang Chengzhi, Liu Yifan, Zhang Xiaoqing, Liu Peimin, Li Dongdong

**Affiliations:** a Henan University of Chinese Medicine, Zhengzhou, China; b The Second Affiliated Hospital of Henan University of Chinese Medicine, Zhengzhou, China; c The First Affiliated Hospital of Zhengzhou University, Zhengzhou, China.

**Keywords:** natural products, tumor-associated macrophages, tumor immunity

## Abstract

As an important innate immune cell in the body, macrophages have a strong ability to phagocytic tumor cells and maintain the innate immune response. Tumor-associated macrophages play a more prominent role in regulating tumor immunity and are currently an important target of antitumor immunity. As a new type of antitumor therapy, tumor immunotherapy has great potential, combined chemotherapy, targeting and other therapeutic means can significantly enhance the antitumor therapy effect. At present, a number of natural products have been proved to have significant immunomodulatory and antitumor effects, and have become a hot field of antitumor immunity research. Studies have found that a variety of natural products, such as polysaccharides, flavonoids, saponins, lactones, and alkaloids, can induce the polarization of M1 macrophages, inhibit the polarization of M2 macrophages, and regulate the expression of immune-related cytokines by targeting specific signaling pathways to enhance the killing effect of macrophages on tumor cells and improve the tumor immune microenvironment, and finally better play the antitumor immune function. In this paper, by summarizing the research results of the specific mechanism of natural products targeting tumor-associated macrophages to exert antitumor immunity in recent years, we discussed the aspects of natural products targeting tumor-associated macrophages to enhance antitumor immunity, in order to provide a new research idea for tumor immunotherapy and further improve the effectiveness of clinical antitumor therapy.

## 1. Introduction

As an important innate immune cell in the body, macrophages have a strong ability to phagocytic tumor cells and maintain the innate immune response. Tumor-associated macrophages play a more prominent role in regulating tumor immunity and are currently an important target of antitumor immunity. In this paper, we discussed the aspects of natural products targeting tumor-associated macrophages to enhance antitumor immunity, in order to provide a new research idea for tumor immunotherapy and further improve the effectiveness of clinical antitumor therapy.

According to the latest global Cancer statistics report, there are about 19.3 million new cancer cases and about 10 million cancer deaths worldwide in 2020, and it is expected that the global cancer cases will be close to 30 million around 2040.^[1]^ However, due to the large population base in China, the incidence and mortality of cancer are the first in the world. It has become the main cause of death from all diseases in our country at present, which seriously endangers the physical and mental health of our people. Conventional treatment in Western medicine, while playing an antitumor role, has side effects such as gastrointestinal reaction, bone marrow suppression and damage to immune function, which is not conducive to the long-term treatment of tumor patients and the improvement of prognosis. Therefore, the search for new antitumor drugs with less toxicity, better efficacy and lower side effects has become the most concerned topic for oncologists around the world.^[2]^ As a new type of antitumor therapy, tumor immunotherapy can effectively activate the tumor immune cycle by targeting specific targets, pathways and mechanisms, so that tumor patients can produce a specific immune response to tumor cells, and finally kill and eliminate tumor cells to inhibit tumor growth and invasion. Studies have found that many natural products have gradually shown significant regulation of macrophage activity, function and polarization process, and have the advantages of a wide range of sources, numerous pharmacological effects and high safety, which are of great significance in antitumor immunity.^[3]^

## 2. Tumor microenvironment and tumor-associated macrophages

**Figure 1.**
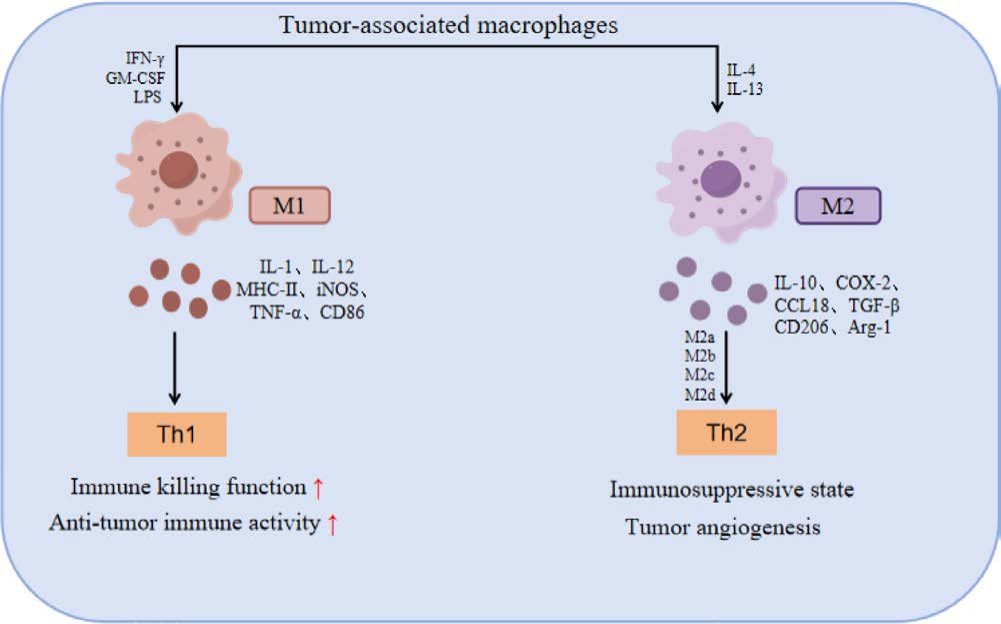
Polarization types of tumor-associated macrophages.

The occurrence and development of malignant tumors are closely related to tumor microenvironment, which is mainly composed of a variety of cells such as tumor cells, immune cells, fibroblasts, etc, as well as various cytokines, and each component has common influence and interaction. Ultimately, it affects the occurrence, development and prognosis of tumors.^[4]^ The macrophages in tumor microenvironment (TME) are also known as tumor-associated macrophages (TAM), which are heterogeneous cells that can promote Tumor cell proliferation and invasion, induce tumor angiogenesis, and affect immune microenvironment.^[5]^ In recent years, more and more studies have shown that TAM is closely related to the immune regulation of a variety of malignant tumors, and it has become an important target for tumor immunotherapy.^[6]^ TAM always changes dynamically with the progression of tumor. According to the difference in function and secreted cytokines, TAM can be divided into M1 type and M2 type^[7]^ (Fig. 1). Some helper T cell 1 cytokines such as IFN-γ, tumor necrosis factor-α (TNF-α), and granulocyte-macrophage colony-stimulating factor, can induce the production of classically activated M1 macrophages, and M1 mainly participates in the helper T cell 1-mediated immune response pathway. Through the release of interleukin (IL)-6, IL-12, IL-23, IL-1β and other immune-related cytokines and ROS, NO, inducible nitric oxide synthase (iNOS) and other related immune response molecules, play a powerful antitumor, clear apoptotic cells, and necrotic tissue ability. At this time, macrophages play an important role in tumor inhibition.^[8]^ In the early stage of tumorigenesis, some pro-inflammatory factors in TME can promote the recruitment and polarization of M1-TAM, thus producing cytotoxic factors, phagocytosis and destruction of tumor cells, and releasing pro-inflammatory factors to exert antitumor effects.^[9]^ The Th2 cytokines such as IL-4, IL-13, IL-10, and transforming growth factor-β (TGF-β), can induce M2-type macrophages, and M2 is mainly involved in Th2-mediated alternative response activation. M2 macrophages can be divided into M2a, M2b, M2c, and M2d subtypes according to their functions. Among them, M2a is induced by IL-4/IL-13 and secretes IL1-Ra, arginase-1 (Arg-1), TGF-β, CCL17, and CCL18, which inhibit M1-TAM polarization and promote tumor cell growth and metastasis. The M2b type is mainly induced by some immune complexes and LPS. Through the release of immune cytokines TNF-α, IL-6, and IL-10, it has high expression of major histocompatibility complex Ⅱ, CD80 and CD86, which has antigen presenting function and plays an important role in immune regulation. M2c type is induced by glucocorticoids and IL-10, and can efficiently express IL-10, TGF-β, CCL16, CCL18, mer tyrosine kinase, etc, which has obvious immunosuppressive effect. M2d type is co-induced by TLR agonists and adenosine receptor agonists, and can promote tumor angiogenesis and immune escape of tumor cells through the secretion of vascular endothelial growth factor (VEGF), IL-10, TGF-β, etc, with significant pro-tumor effect.^[10]^ Therefore, different regulatory effects on different phenotypes of M2 macrophages are the key to improving TAM ratio, enhancing TAM function and activity, and enhancing antitumor immune response. In addition, M2-TAM also has a regulatory effect on TIME, and can secrete cytokines (VEGF, IL-4, IL-10, platelet-derived growth factor), chemokines (c-c motif chemokine ligand 2, CCL17), and proteases (COX-2, Arg1, matrix metalloproteinase [MMP]) to inhibit the antitumor immune response mediated by T cells. Reduce the killing function of CD8^+^T lymphocytes and CD4^+^T lymphocytes to tumor cells; it can also inhibit T cell activation through PD-1/PD-L1 pathway, induce T cell apoptosis, and then immune escape, promote tumor progression and metastasis.^[11]^ With the progression of tumor, the immunosuppressive cytokines IL-4, IL-10, and IL-13 in TME induce the polarization of macrophages towards M2 phenotype, and induce the gradual polarization of M1-TAM into M2-TAM, which will eventually promote the proliferation and invasion of tumor cells, induce immune suppression and enhance tumor drug resistance. At this time, macrophages showed significant tumor promoting effect.^[12]^

## 3. Natural products target TAM to enhance antitumor immunity

**Figure 2.**
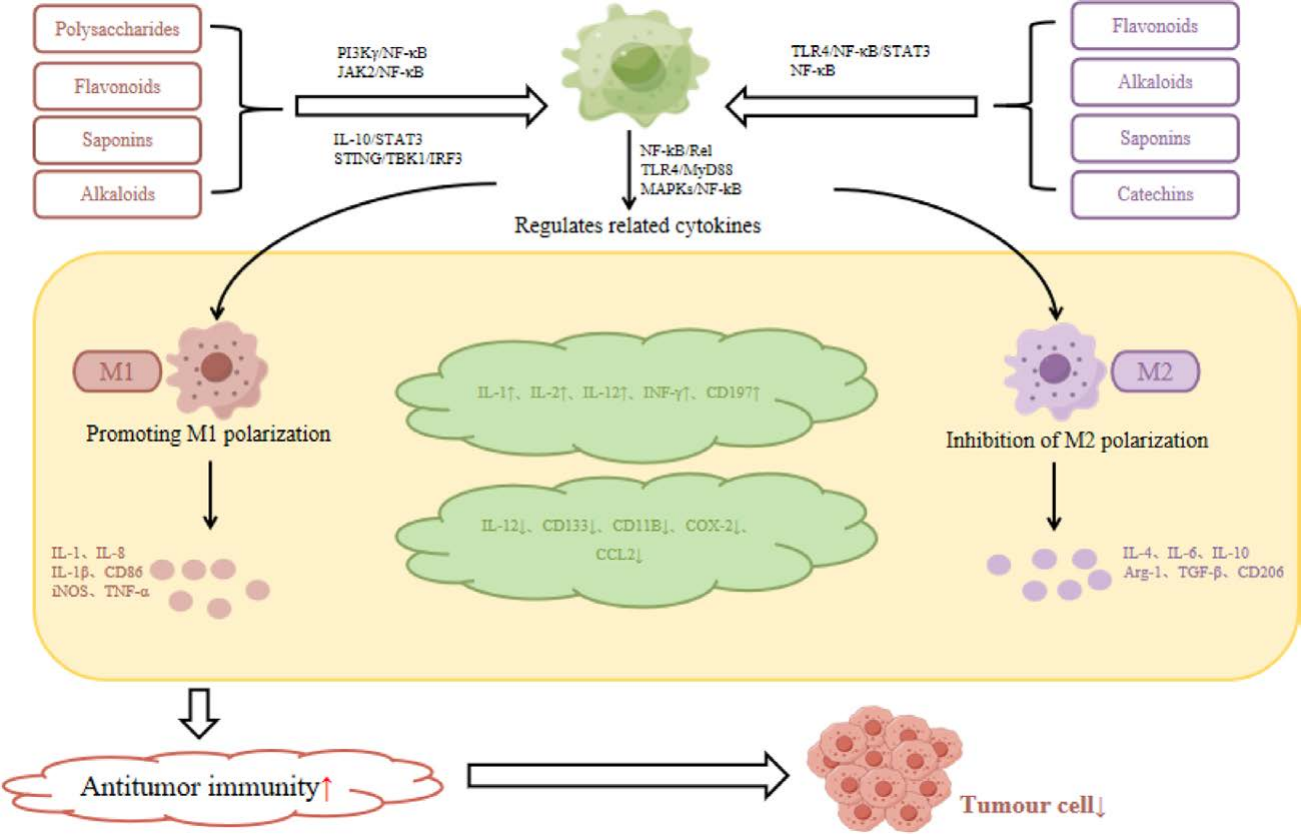
Specific mechanism of antitumor immune effect of natural products targeting tumor-associated macrophages.

TAM has high plasticity and has always affected the occurrence, development and prognosis of tumors. The antitumor treatment strategy of regulating M1/M2 polarization has great potential. M1-type macrophages mainly secrete NO, ROS, TNF-α, IL-1, IL-6, IL-12, L-23, and other cytokines to enhance the immune killing effect of M cells and improve antitumor immunity.^[13]^ M2 macrophages mainly secrete a large number of pro-angiogenic factors, such as VEGF-A, epidermal growth factor, TNF-α, etc, to promote tumor angiogenesis and tumor cell proliferation and migration.^[14]^ At the same time, it can also promote c-c motif chemokine ligand 2, IL-6, IL-10, CD16 and CD163, CD204, CD200R, and other specific cytokines. These factors promote TME to generate negative immune feedback by activating PI3K/Akt/mTOR, IL-6/STAT3, JAK1/STAT1/NF-κB/Notch1^[15–17]^ signaling pathways, thereby inhibiting the innate immune response and promoting tumor proliferation and invasion. It can inhibit the innate immune response and promote the proliferation and invasion of tumor. Studies have found that natural products have the advantages of high safety, multiple targets and wide pharmacological effects, which can regulate TAM, affect the expression of related cytokines, improve the ratio of M1/M2 macrophages, promote the phenotype polarization of TAM to M1, improve TME, correct the immunosuppressive state, and enhance the inherent immune response of tumor patients. Improve the effectiveness of inhibiting tumor growth and metastasis (Fig. 2). In this review, a variety of natural products such as polysaccharides, flavonoids, saponins, lactones, alkaloids, coumarins, catechinins, etc, regulate macrophage polarization and related cytokines by targeting specific signaling pathways, and finally exert antitumor immune effects.

### 3.1. Polysaccharides

Polysaccharides have the characteristics of high active ingredients and extensive pharmacological effects. In terms of immune regulation, polysaccharides natural products have multi-channel and multi-level regulatory effects on the immune system, especially for tumor-related macrophages. A large number of studies have shown that the immunomodulatory effects of polysaccharide natural products on tumor-associated macrophages are mainly achieved by adjusting M1 and M2 macrophage-related markers and cytokines, improving the activity of macrophages, and enhancing the phagocytosis and killing ability of macrophages. Therefore, in-depth study of the relationship between polysaccharide natural products and antitumor immunity has become the key to explore new effective means of tumor therapy. Safflower polysaccharide is a kind of polysaccharide compound extracted from Safflower, which has the pharmacological effect of regulating immunity. Studies have found that Safflower polysaccharide can significantly increase the expression levels of TNF-α and NO, thereby inducing Raw 264.7 macrophages to polarize towards M1 phenotype, and ultimately play a role in inhibiting the growth and metastasis of mouse colorectal cancer induced by AOM/DSS.^[18]^ Another experiments have proved that SPPC could inhibit the expression levels of IL-10 and TGF-β, thereby increasing the proportion of M1 macrophages and enhancing antitumor immunity.^[19]^ Camphor polysaccharides come from the natural plant camphor, study have found that Camphor polysaccharide can reduce the expression levels of IL-6, IL-10, and TGF-β in tumor-bearing mice, thereby promoting the transformation of macrophages to M1 phenotype and ultimately enhancing antitumor immunity.^[20]^ Pachymaran is the main active component of Poria cocos, and a lot of clinical and pharmacological studies have found that Pachymaran has a good antitumor effect, Jia W et al^[21]^ found that pachymaran can promote the massive release of IL-1β, TNF-α, and iNOS by down-regulating the JAK2/NF-κB signaling pathway, increase the expression of CD40, CD86, and CD23, and thus induce the transformation of macrophages to M1 phenotype, and ultimately enhance the killing effect on bladder cancer cells. Polyporus polysaccharide is a polysaccharide compound from porus, which has the pharmacological effect of enhancing immune function and antitumor, Liu team found that polyporus polysaccharide could increase the expression levels of iNOS, TNF-α, and IL-6, and increase the expression of CD86, a marker of M1 macrophages, thus promoting the polarization of M1 macrophages and enhancing antitumor immunity.^[22]^ Astragalus polysaccharide is the most important active ingredient of Astragalus and has significant antitumor immunity, Bamodu O A found in vivo and in vitro studies that Astragalus polysaccharide can induce polarization of M1 macrophages and reduce the expression of CD206, a marker of M2 macrophages, thereby inhibiting the growth and metastasis of non-small cell lung cancer.^[23]^ A study by K Y et al also showed that Astragalus polysaccharide can release more NO and TNF-α by activating toll-like receptor 4 (TLR4) receptor and NF-kB/Rel signaling pathway, thus playing an antitumor immune role.^[24]^ Similarly, an in vitro experiment found that Astragalus polysaccharide can significantly enhance the phagocytic function of mouse macrophages and increase the production of IL-2, TNF-α and interferon-γ (INF-γ), thus enhancing antitumor immunity.^[25]^ In a study of breast cancer mice, Li W et al found that Astragalus polysaccharide could activate RAW264.7 macrophages, increase the expression of NO and TNF-α, and thus enhance the inhibitory effect on MCF-7 breast cancer cells.^[26]^ In another study on co-culture of cervical cancer tumor cells with human peripheral blood mononuclear cells, Shokati E et al found that Astragalus polysaccharide can enhance immune response by reducing the concentration and expression of IL-10 and TGF-β, thus inhibiting the growth of cervical cancer tumor cells. Notch signaling pathway is closely related to the occurrence and development of non-small cell lung cancer. Wei W et al found that Astragalus polysaccharide can promote the production of IL-6, TNF-α, and iNOS and other cytokines through Notch signaling pathway, thus enhancing the immune killing effect on tumor cells of non-small cell lung cancer.^[27]^ Similarly, the activity of TLR4/MyD88 signaling pathway also affects the occurrence and development of tumors. Zhou L concluded in vivo and in vitro studies that Astragalus polysaccharides can promote the secretion and expression of macrophage-related cytokines NO, TNF-α, IL-1β, and IL-6 by regulating the TLR4/MyD88 signaling pathway. Thus playing an antitumor immune role.^[28]^ In an in vivo experiment on tumor-bearing mice, Yang B et al found that Astragalus polysaccharide could increase the production and expression of NO, IL-1β, IL-6, and TNF-α in tumor-bearing mice, thereby enhancing the killing effect on tumor cells of tumor-bearing mice and inhibiting tumor growth and metastasis.^[29]^ Chinese yam and tremella tremella, as the typical representatives of the homology of medicine and food, have obvious immunomodulatory effects due to their main active ingredients. Studies have shown that yam polysaccharide can promote the expression of TNF-α, IL-6, and monocyte chemoattractant protein-1, and also promote macrophages to secrete more NO, thus inducing macrophages to polarize towards M1 phenotype.^[30]^ Tremella polysaccharide can promote the polarization of macrophages towards M1 phenotype, enhance antitumor immunity, and thus induce rapid apoptosis of B16 melanoma cells.^[31]^ Seabuckthorn polysaccharide is a natural polysaccharide derived from natural plant seabuckthorn fruits. Sun S et al found that sea buckthorn polysaccharide can increase the release of NO and up-regulate the expression levels of TNF-α, IL-2, IL-4, and IL-6, thus enhancing antitumor immunity.^[32]^ *Ganoderma lucidum* polysaccharide is the main active component of *G lucidum* in exerting pharmacological effects. Studies have found that *G lucidum* polysaccharides can regulate MAPKs and NF-kB signaling pathways by affecting Dectin-1 and TLR2 receptors, increase the expression levels of cytokines NO, ROS, and TNF-α, and enhance the phagocytic function of macrophages, and finally play a role in inhibiting tumor growth.^[33]^ Psyllium polysaccharide is a natural polysaccharide from Psyllium seed. Many pharmacological studies have shown that Psyllium polysaccharide plays an important role in the regulation of immunity. Recent studies have found that plantago polysaccharide can induce the activation of J774 macrophages, increase the release of NO and TNF-α, and thus enhance the antitumor effect.^[34]^ In a study on extraction, hydration and pharmacology of aconite polysaccharide, Yang X found that a water-extracted aconite polysaccharide could promote the phagocytosis of RAW264.7 macrophages and induce the production of a large amount of NO, IL-6, IL-1, and TNF-α, thus providing antitumor immunity.^[35]^ Mushroom polysaccharide is a natural product extracted from *G lucidum*. Wang W J found that mushroom polysaccharide can adjust the proportion of M1/M2 macrophages, increase the expression levels of IL-12 and IFN-γ, and reduce the expression levels of IL-10, COX-2, and TGF-β, thereby improving the state of immunosuppression and playing an antitumor immune role.^[36]^ To sum up, natural products such as Safflower polysaccharide, SPPC, Camphor polysaccharide, Pachymaran, Astragalus polysaccharide, Yam polysaccharide, Tremella polysaccharide, Sea buckhorn polysaccharide, *G lucidum* polysaccharide, Planteola polysaccharide and Mushroom polysaccharide can regulate JAK2/NF-κB, NF-kB/Rel, TLR4/MyD88, Notch, MAPKs/NF-kB signals Pathway, increase the expression of M1-related cytokines (IL-1, IL-6, TNF-α, INF-γ, etc), chemotactic protein monocyte chemoattractant protein-1 and specific markers CD80, CD86, CD40, CD23, and promote the release of NO and iNOS. Decrease the expression of M2-related cytokines (IL-6, IL-10, TGF-β, etc) and markers CD206, CD163, etc, thereby inducing M1-TAM polarization, inhibiting M2-TAM polarization, improving M1/M2 ratio, enhancing TAM immune function, enhancing killing activity, and finally playing antitumor immune role, inhibit the growth and metastasis of NSCLC, colorectal cancer, bladder cancer, cervical cancer, breast cancer, melanoma, etc.

### 3.2. Flavonoids

Flavonoids are a kind of common natural compounds in nature, with strong biological activities and extensive pharmacological effects. The latest pharmacological studies have shown that flavonoids can play an antitumor role by regulating the immune system and enhancing the immune function of the body. Baicalin is one of the main active compounds extracted from scutellaria baicalensis, which has significant antitumor effect. He S et al found that baicalein can increase the expression of M1 macrophage-related factors TNF-α, IL-1β, chemokine (C-X-C motif) ligand 9, and CXCL10 by regulating the PI3Kγ/NF-κB signaling pathway, thereby inducing M1 macrophage polarization and inhibiting the proliferation and invasion of breast cancer and melanocyte.^[37]^ In a study on xenotransplantation models of non-small cell lung cancer, Kang H found that puerarine can increase levels of CD197, iNOS, CD40, IFN-γ, TNF-α, and IL-12 and decrease the expression of CD206, Arg-1, CD163, IL-10, IL-4, and TGF-β by inhibiting the MEK/ERK 2/1 signaling pathway, thereby promoting M1 phenotype polarization, inhibiting M2 phenotype polarization, and ultimately inhibiting the growth and metastasis of tumor cells in non-small cell lung cancer xenotransplantation model.^[38]^ Silybin is a natural flavonoid derived from milk thistle seeds. In a study on tumor-bearing mice, Forghani P found that silybin could inhibit the polarization of M2 macrophages, thereby enhancing antitumor immunity and reducing tumor volume in tumor-bearing mice.^[39]^ Glycyrrhiza is a kind of traditional Chinese medicine which is widely used in antitumor treatment. Glycyrrhiza flavone is one of the main active components of glycyrrhiza. Studies have shown that total glycyrrhiza flavones can indirectly play an antitumor immune role by reducing the phosphorylation level of STAT6, blocking IL-4/IL-13 induced activation of STAT6, inhibiting the polarization of M2 macrophages.^[40]^ In another study on pancreatic cancer tumor cells, Zheng X et al found that icariin can prevent macrophages from polarizing toward M2 phenotype and reduce the number of M2 macrophages by inhibiting the IL4/STAT6 signaling pathway, thus delaying the proliferation and invasion of pancreatic cancer tumor cells.^[40]^ In summary, natural flavonoid products such as Baicalein, Puerarin, Silybin, Glycyrrhiza flavones, and Icariin can regulate of PI3Kγ/NF-κB, MEK/ERK 2/1, and IL4/STAT6 signaling pathways, increase the expression of TNF-α, IL-1β, IL-12, chemokine (C-X-C motif) ligand 9, CXCL10, CD197, reduce the expression of Arg-1, IL-10, IL-4, TGF-β, CD163, CD206, etc, promote M1 polarization, inhibit M2 polarization, and ultimately play an antitumor role, inhibit the growth and metastasis of breast cancer, melanoma, NSCLC, pancreatic cancer, etc.

### 3.3. Saponins

Natural product saponins can enhance the immunity of the body and play an antitumor role by means of multiple pathways, multiple targets and multiple mechanisms. It has been found that saponins have natural immunomodulatory activities and can play an antitumor immune role by regulating the activity and polarization ratio of tumor-associated macrophages. Mathiyalagan R et al found that ginsenosides can significantly down-regulate the expression of CD206, a marker of M2 macrophages, and increase the expression of CD16 and CD32 in M1 macrophages, thereby transforming M2 macrophages into M1 phenotypes and ultimately enhancing the antilung cancer effect.^[41]^ Additionally, Li H et al found that ginsenosides can induce the transformation of M2 macrophages to M1 phenotype and reduce the expression of MMP2 and MMP9, thus inhibiting the growth and metastasis of lung cancer cells.^[42]^ Zonoside is a commonly used antitumor Chinese herbal medicine, and Zonoside VII is the main active component to play antitumor role. Yu J et al found that Zonoside VII can increase the expression level of iNOS by activating STING/TBK1/IRF3 signaling pathway, thus inducing macrophages to polarize towards M1 phenotype. At the same time, the number of invasive LLC and A549 co-cultured with M1 macrophages can be directly reduced, and the proliferation and invasion of lung cancer cells can be inhibited.^[43]^ Another study found that astragaloside can inhibit the growth and migration of liver cancer cells by regulating the TLR4/NF-κB/STAT3 signaling pathway, reducing the expression of CD206 and inhibiting the polarization of M2 macrophages.^[44]^ Saikosaponin A is isolated from the dried roots of bupleurum, and has antitumor activity. Zhao X found in A study on the anti-breast cancer mechanism of saikosaponin A that saikosaponin A can increase the expression levels of IL-12 and IFN-γ, and reduce the expression levels of IL-4 and IL-10, thereby inhibiting the growth and metastasis of breast cancer cells.^[45]^ In conclusion, saponins such as Ginsenoside, Polyphyllin VII, Astragaloside and Saikosaponin A can regulate the STING/TBK1/IRF3 and TLR4/NF-κB/STAT3 signaling pathways, improve the expression of IL-12, IFN-γ, iNOS, CD16, CD32, and reduce the expression of IL-4, IL-10, CD206, MMP2, MMP9, improve the M1-TAM/M2-TAM ratio, and finally inhibit the progression of lung cancer, liver cancer, breast cancer, etc.

### 3.4. Lactones

Triptolide is an epoxide diterpene lactone compound extracted from the roots, leaves, flowers and fruits of triptolide, which has immunomodulatory effects. A study on ovarian cancer confirmed that triptolide combined with cisplatin could significantly down-regulate the expression of CD206 and CD31 and reduce the number of M2 macrophages, thereby enhancing antitumor immunity.^[46]^ β-Elemene is the main active component from aurantium. Yu X et al found that β-elemene can reduce the expression of M2 marker Arg-1, thus inhibiting the polarization of M2 macrophages, and ultimately inhibiting the growth, migration and invasion of Lewis lung cancer tumor cells.^[47]^ Dihydroartemisinin is a derivative of artemisinin, which has antitumor activity. Chen R et al found that dihydroartemisinin can prevent the polarization of M2 macrophages by blocking the activation of STAT3 pathway, thus inhibiting the growth and migration of Fadu cells and Cal-27 cells in head and neck squamous cell carcinoma.^[48]^ Therefore, lactones mainly inhibit M2-TAM, such as triptolide, β-elemene, and dihydroartemisinin, which can significantly down-regulate the expression of CD206, CD31, and Arg-1, thereby inhibiting M2 polarization, reversing the immunosuppressive state, and ultimately inhibiting the growth of lung cancer and head and neck squamous cell carcinoma.

### 3.5. Alkaloids

Berberine is an alkaloid compound isolated from *Coptis chinensis*, and it is also the main effective component of *C chinensis* to play antitumor role. According to the study of Piao M, berberine can reduce the expression of IL-12, a marker of M2 macrophage, and up-regulate the expression of IFN-γ, a marker of M1 macrophage. This can induce the transformation of M2 macrophages into M1 macrophages and inhibit the migration and invasion of colorectal tumor cells.^[49]^ Another study found that matrine could inhibit the polarization of M2 macrophages RAW264.7 by up-regulating the expression of E-cadherin and down-regulating the expression of N-cadherin, vimentin and Snail, and reduce the secretion and expression of IL-4, IL-10, and Arg-1. Finally, it can inhibit the metastasis of Lewis lung cancer cells in mice.^[50]^ In consequence, Berberine can promote M1-TAM polarization by up-regulating the expression of IFN-γ. Matrine inhibits M2-TAM polarization by down-regulating the expression of IL-4, IL-10, and Arg-1, which can ultimately enhance TAM immune function and inhibit the growth of lung cancer and colorectal cancer.

### 3.6. Coumarins

Osthole is a natural compound extracted from the fruit of osthole, which is also a very important natural active ingredient. In a study of pancreatic cancer tumor cells, Wang B et al found that Osthole could reduce the expression of C/EBPB, inhibit the phosphorylation of STAT6, and thus reduce the expression of TGF-β, CCL22, CD206, CD11B, and MRC1, block the activation of M2 macrophages and inhibit the polarization of M2 macrophages. At the same time, it can also reduce the number of M2 macrophages in the spleen of pancreatic cancer mice, and significantly inhibit the proliferation and invasion of pancreatic cancer tumor cells.^[51]^

### 3.7. Catechins

NF-κB signaling pathway is closely related to the occurrence and development of a variety of tumors. In a study on gynecological tumors, Huang Y J et al confirmed that epigallocatechin gallate inhibited the polarization of macrophages into M2 phenotype by inhibiting NF-κB signaling pathway, reducing the expression of IL-6 and TGF-β, increasing the expression of TNF-α, and inhibiting the polarization of macrophages into M2 phenotype. Finally, it plays an inhibitory role in anti-endometrial cancer, breast cancer and ovarian cancer.^[52]^

### 3.8. Polyphenols

Resveratrol is a kind of polyphenol organic compound mainly derived from veratrol. Many in vitro and animal experiments have shown that resveratrol has antitumor effects. Kimura Y et al found that resveratrol can significantly inhibit the polarization and activation of M2 macrophages, thereby inhibiting the metastasis of osteosarcoma to the lung and liver and preventing the formation of lymphatic vessels.^[53]^ In another study, Sun L et al found that resveratrol could inhibit the polarization of M2 macrophages in mice with lung cancer by reducing the activity of STAT3, thus exerting its tumor-shrinking effect on mice with lung cancer.^[54]^ Pterostilbene is an active ingredient from rosewood, blueberry, grape and other plants. Studies have shown that it can play an antitumor role. Huang WC et al found that Pterostilbene can reduce the expression levels of MUC1 and e-cadherin, the genes related to M2 macrophage polarization, and at the same time reduce the amount of CD133 and Sox2, thereby inhibiting the polarization of M2 macrophages and ultimately inhibiting the proliferation and migration of tumor cells A549 and H441.^[55]^ In a word, polyphenols play a regulatory role in TAM mainly by inhibiting M2 polarization; resveratrol and pterostilbene can inhibit M2-TAM polarization and decrease the number of M2-TAM by decreasing the expression of MUC1, E-Cadherin, CD133 and Sox2, and finally inhibit the metastasis of osteosarcoma, A549 and H441 cells.

### 3.9. Other categories

Dandelion is one of the commonly used antitumor traditional Chinese medicine for clearing heat and detoxification, and its active components have significant antitumor effects. Deng XX et al found that dandelion extract could inhibit the IL-10/STAT3/PD-L1 signaling pathway, promote the expression of TNF-α, IL-8, and iNOS, reduce the expression of IL-10, CD206, and TGF-β, and induce the polarization of macrophages from M2 phenotype to M1 phenotype. Finally, it inhibits the proliferation and invasion of triple-negative breast cancer tumor cells.^[56]^ Dual-drug chemotherapy regimen containing DDP is a commonly used chemotherapy regimen for lung cancer. Chen Y et al found that ginseng and Astragalus water extract combined with DDP can increase the expression levels of IL-12, TNF-α, and IFN-γ, and decrease the expression levels of IL-10 and TGF-β, thus adjusting the ratio of M1/M2 macrophages. Inhibit the growth and metastasis of Lewis lung cancer.^[57]^ Therefore, Dandelion extract can induce M2-TAM polarization to M1-TAM by inhibiting IL-10/STAT3/PD-L1 signaling pathway. *Ginseng astragalus* water extract can regulate the expression of TAM-related cytokines to improve the M1/M2 ratio. Ultimately inhibiting the progression of breast and lung cancer.

## 4. Discussion

**Figure 3.**
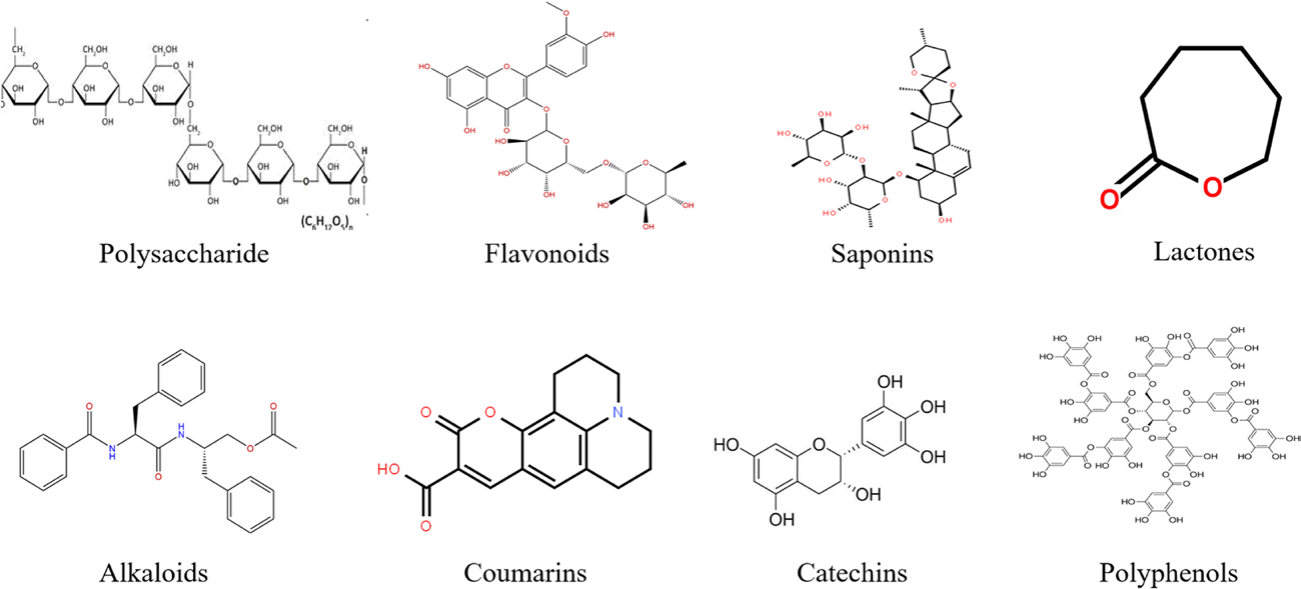
Molecular structure formula of natural products targeting TAM. TAM = tumor-associated macrophages.

The high incidence and high mortality of malignant tumors make them the first killer that endangers people’s health. With the wide application of tumor immunotherapy, antitumor therapy has achieved remarkable clinical effect. The natural product (Fig. 3) can enhance the activity and function of macrophages by targeting and regulating tumor-related macrophages, induce the polarization of macrophages towards M1 phenotype, increase the number and expression of immunoactive cytokines, and ultimately play a better antitumor immune role (Table 1). Although some achievements have been made in the relevant studies on the targeted regulation of TAM by natural products to play an antitumor immune role, there are still some problems: (1) at present, the researches on the regulation of TAM by natural products are mainly animal experiments and in vitro experiments, and the researches on the direct effect of TAM on human body are relatively few; (2) natural products can play an antitumor immune role by regulating macrophages, but their composition is complex, difficult to extract, and purity level is low, so it is still difficult to carry out relevant mechanism research; (3) some natural products exert antitumor immunity by targeting macrophages, and macrophages are only a part of the whole tumor immune microenvironment, not the whole link of antitumor immunity, and their specific mechanisms need to be further explored and clarified; (4) targeting of natural products to macrophages for antitumor immunity may involve a specific signaling pathway, which still needs to be further studied for better labeling and elaboration.

**Table 1:** Summary of specific mechanisms of antitumor immunity of macrophages targeted by natural products.

Types of natural products	Natural product	Source	Regulating the mechanism of action of macrophages	References
Polysaccharides	Safflower polysaccharide	Safflower	Increase the expression of TNF-α and NO, promote the polarization of Raw 264.7 macrophages to M1 phenotype.	[18]
	SPPC	Centipede	Inhibition of IL-10, the expression of TGF-β, increase proportion of M1 macrophages.	[19]
	Camphor polysaccharide	Camphor	Decrease the expression of IL-6, IL-10 and TGF-β, and promote the transformation of macrophages to M1 phenotype.	[20]
	Pachymaran	Poria cocos	Promote the expression of IL-1β, TNF-α and iNOS by down-regulating JAK2/NF-κB signaling pathway.	[21]
	Polyporus polysaccharide	Polyporus	Increase the expression of iNOS, TNF-α, IL-6 and CD86, and promote the polarization of M1 macrophages.	[22]
	Astragalus polysaccharide	Astragalus	Promote the polarization of M1 macrophages; reduce the M2 macrophage marker CD206.	[23]
			Release more NO and TNF-α by activating the TLR4 receptor and the NF-kB/Rel signaling pathway.	[24]
			Increase the production of IL-2, TNF-α and INF-γ.	[25]
			Activate RAW264.7 macrophages; increase the expression of NO and TNF-α.	[26]
			Decrease the concentration and expression of IL-10 and TGF-β.	[58]
			Promote the production of IL-6, TNF-α and iNOS by regulating the Notch signaling pathway.	[27]
			Promote the expression of NO, TNF-α and IL-1β/IL-6 by regulating the TLR4/MyD88 signaling pathway.	[28]
			Increase the expression of NO, IL-1β, IL-6 and TNF-α in tumor-bearing mice.	[29]
	Chinese yam polysaccharide	Chinese yam	Promote the expression of TNF-α and IL-6, and induce the polarization of macrophages towards M1 phenotype.	[30]
	Tremella polysaccharide	Tremella	Promote the polarization of macrophages towards M1 phenotype.	[31]
	Seabuckthorn polysaccharide	Seabuckthorn	Increase the release of NO and up-regulate the expression of TNF-α, IL-2, IL-4, and IL-6.	[32]
	*Ganoderma lucidum* polysaccharide	*Ganoderma lucidum*	Increase the expression of NO, ROS, and TNF-α by regulating MAPKs and NF-kB signaling pathways.	[33]
	Plantain acid polysaccharide	Plantain	Induce the activation of J774 macrophages; increase the expression of NO and TNF-α.	[34]
	Aconite polysaccharide	Aconite	Promote phagocytosis of RAW264.7 macrophages and produce a large amount of NO, IL-6, IL-1 and TNF-α.	[35]
	Mushroom polysaccharide	Mushroom	Increase the expression of IL-12 and IFN-γ; decrease the expression of IL-10, COX-2 and TGF-β.	[36]
Flavonoids	Baicalein	*Scutellaria baicalensis*	Increase the expression of TNF-α and IL-1β and induce the polarization of M1 macrophages by regulating the PI3Kγ/NF-κB signaling pathway.	[37]
	Puerarin	Kudzu vine root	Promote the polarization of macrophages towards M1 phenotype; decease the expression of M2-related tumor promoting cytokines.	[38]
	Silybin	*Silybum marianum*	Inhibition of polarization of M2 macrophages.	[39]
	Total licorice flavones	Liquorice	Inhibit the polarization of M2 macrophages by decreasing the level of STAT6 phosphorylation and blocking IL-4/IL-13 induced STAT6 activation.	[40]
	Icariin	Epimedium	Inhibit the polarization of M2 macrophages by down-regulating IL4/STAT6 signaling pathway.	[59]
Saponins	Ginsenoside	Ginseng	Decrease the expression of CD206 and increase the expression of CD16 and CD32.	[41]
			Induce M2 macrophages to change to M1 phenotype, and decrease the expression of MMP2 and MMP9.	[42]
	Polyphyllin VII	*Rhizoma paridis*	Increase the expression of iNOS, and induce the polarization of M1 phenotype by activating STING/TBK1/IRF3 signaling pathway.	[43]
			Directly reduce the number of aggressive LLC and A549 co-cultured with M1 macrophages.	
	Astragaloside	Astragalus	Regulate TLR4/NF-κB/STAT3 signaling pathway, reduce CD206 expression, inhibit the polarization of M2 macrophages.	[44]
	Saikoside	*Radix bupleuri*	Increase the expression of IL-12 and IFN-γ; decrease the expression of IL-4 and IL-10.	[45]
Lactones	Triptolide	Thunder god vine	Combined with cisplatin can reduce the expression of CD206 and CD31, and reduce the number of M2 macrophages.	[46]
	β-Elemene	*Radix curcumae*	Decrease the expression of M2 marker Arg-1 and inhibited the polarization of M2 macrophages.	[47]
	Dihydroartemisinin	*Artemisia apiacea*	Inhibition of IL-4/IL-6 induced M2 macrophage polarization by inhibiting STAT3 pathway.	[48]
Alkaloids	Berberine	*Coptis chinensis*	Decrease IL-12 expression, increase IFN-γ expression, induce M2 macrophages to M1 transformation.	[49]
	Matrine	Radix sophorae flavescentis	Increase the expression of E-cadherin, and decrease the expression of N-cadherin, vimentin and Snail.	[50]
			Inhibit the polarization of M2-type macrophages and reduce the expression of IL-4, IL-10 and Arg-1.	
Coumarins	Osthole	Common Cnidium fruit	Decrease the expression of TGF-β, CCL22, CD206, CD11B and MRC1, and inhibit the polarization of M2 macrophages.	[51]
			Decrease the number of M2 macrophages in spleen of pancreatic cancer mice.	
Catechins	Gallatechin gallate	Acacia catechu	Reduce the expression of IL-6 and TGF-β and increase the expression of TNF-α by inhibiting the NF-κB signaling pathway.	[52]
Polyphenols	Resveratrol	Black false hellebore	Inhibition of polarization and activation of M2 macrophages.	[53]
			Inhibition of M2 macrophage polarization by reducing STAT3 activity.	[54]
	Pterostilbene	Padauk	Decrease the expression of M2 macrophage polarization related genes mucin 1 (MUC1) and E-cadherin.	[55]
			Reduce the amount of CD133 and Sox2; inhibit M2 macrophage polarization.	
Other categories	Dandelion extract	Dandelion	Promote the expression of TNF-α and iNOS; reduce the expression of IL-10 and TGF-β.	[56]
	Ginseng and Astragalus water extract	Ginseng and Astragalus	Combined with cisplatin increase the expression of IL-12, TNF-α and IFN-γ, and decrease the expression of IL-10 and TGF-β.	[57]

MMP = matrix metalloproteinase.

## 5. Future and outlook

The future research on the antitumor immune effect of natural products targeting TAM should not only improve the extraction and purification technology, but also explore the specific mechanism of regulating the specific signal pathway of TAM and increase the precision of TAM targeting, but also carry out a large number of clinical studies to increase the clinical application of related studies, provide more support and validation of clinical data and a solid theoretical basis for the research and development of new drugs for clinical antitumor therapy. In addition, in future studies, advanced technologies such as metabolomics and single-cell sequencing can be used to screen natural products, develop new natural product-related delivery technologies and strategies, expand the combination therapy of natural products with existing clinical antitumor drugs, and develop new drugs to promote TIME remodeling based on different TAM targets, improve the efficacy and safety of natural products in antitumor therapy.

## Author contributions

**Conceptualization:** Wang Chengzhi, Liu Yifan.

**Data curation:** Wang Chengzhi, Liu Yifan, Li Dongdong.

**Formal analysis:** Wang Chengzhi, Liu Yifan, Li Dongdong.

**Funding acquisition:** Wang Chengzhi, Liu Yifan, Li Dongdong.

**Investigation:** Wang Chengzhi, Liu Yifan, Liu Peimin, Li Dongdong.

**Methodology:** Wang Chengzhi, Liu Yifan, Liu Peimin, Li Dongdong.

**Project administration:** Wang Chengzhi, Liu Yifan, Liu Peimin.

**Resources:** Wang Chengzhi, Liu Yifan, Liu Peimin.

**Software:** Wang Chengzhi, Liu Yifan, Liu Peimin.

**Supervision:** Wang Chengzhi, Liu Yifan, Liu Peimin.

**Validation:** Wang Chengzhi, Liu Yifan, Liu Peimin.

**Visualization:** Wang Chengzhi, Liu Yifan, Zhang Xiaoqing, Liu Peimin.

**Writing – original draft:** Wang Chengzhi, Liu Yifan.

**Writing – review & editing:** Wang Chengzhi, Liu Yifan.
